# Patient complexity does not affect surgical learning curve and clinical outcomes during early experience in robotic assisted coronary surgery

**DOI:** 10.1007/s11701-025-02370-w

**Published:** 2025-05-28

**Authors:** Fabrizio Rosati, Massimo Baudo, Lorenzo Di Bacco, Wouter Oosterlinck, Gianluca Torregrossa, Cesare Tomasi, Francesca Boldini, Claudio Muneretto, Stefano Benussi

**Affiliations:** 1 Division of Cardiac Surgery, ASST Spedali Civili Di Brescia, University of Brescia, P.Le Spedali Civili, 1, 25123 Brescia, Italy; 2 Department of Cardiac Surgery Research, Lankenau Institute for Medical Research, Main Line Health, Wynnewood, PA USA; 3 Division of Cardiac Surgery, KU University Hospitals Leuven, Louvain, Belgium; 4 Department of Cardiac Surgery, Lankenau Medical Center, Main Linea Health, Wynnewood, PA USA

**Keywords:** CABG, Minimally-invasive, Robotic surgery, MIDCAB, Learning curve

## Abstract

Adoption of robot-assisted coronary artery bypass grafting (RA-MIDCAB) remains limited due to concerns about learning curves, outcomes, and patient-specific anatomic challenges. This study evaluates our initial single-center experience with RA-MIDCAB. Between December 2022 and June 2024, 52 patients underwent RA-MIDCAB. Inclusion criteria comprised isolated left anterior descending artery (LAD) stenosis or LAD revascularization as part of a hybrid valvular/coronary strategy. Primary endpoints were 30-day mortality, conversion to sternotomy, and graft injury. Operative times and biometric indices (body indices such as body mass index [BMI], Haller Index, and Cardiothoracic Ratio) were analyzed for correlation with learning curve progression and surgical outcomes. Mean age was 68.5 ± 11.5 years, and 82.7% (43/52) were males. Robotic LITA harvesting was successfully completed in 98.1% (51/52) of patients (one patient had a graft injury), with no perioperative mortality. Postoperative complications occurred in 38.5% (20/52), mostly due to atrial fibrillation (19.2%, 10/52) and acute kidney injury (13.5%, 7/52) with no correlation with operative times at logistic regression. According to thoracic indexes, no correlation was found between chest complexity and postoperative complications. Neither EuroSCORE II, BMI nor thoracic indices significantly impacted operative times. Linear regression demonstrated significant reductions in overall surgical and graft-harvesting times across the experience, suggesting improved efficiency. RA-MIDCAB is feasible and safe, even in patients with challenging thoracic anatomy. This early experience demonstrated promising outcomes and significant learning curve improvements, supporting the potential for broader adoption of this technique even in patients unlikely deemed suitable for minimally invasive cardiac revascularization surgery.

**Supplementary Information:**

The online version contains supplementary material available at 10.1007/s11701-025-02370-w.

## Introduction

Several studies showed robot-assisted coronary artery bypass grafting (CABG) is a safe and effective approach for surgical revascularization. This technique facilitates closed-chest minimally invasive harvesting of the internal thoracic arteries, which can be successively anastomosed either endoscopically (TECAB) [1] or through a small mini-thoracotomy under direct vision (RA-MIDCAB) [2]. Over recent years, centers worldwide have expanded the use of a robotic CABG for treating multivessel coronary artery disease often in conjunction with percutaneous coronary intervention (PCI) as a part of hybrid revascularization (HR) strategy [3, 4]. These methods offer the long-term benefits of a surgical revascularization while avoiding the drawbacks associated with midline sternotomy [5]. Despite the promising results, adoption of robotic-assisted CABG remains low in the U.S., although a slight increase has been noted in Europe [6, 7]. The primary barriers to broader acceptance include concerns regarding the need for optimal robotic team training, the steep learning curves associated with robotic surgery, the lack of consistent data about the outcome of HR, and the potential prolonged operative times that may impact clinical results especially at an early stage of the robotic surgical experience [8]. We present our single-center experience with the first 52 cases of RA-MIDCAB. Specifically, we investigated the impact of patient’s body indexes (body mass index [BMI], Haller Index [HI] and Cardiothoracic Ratio [CR]) on safety profile and surgical performance progression in the first all-comers series independently from surgical risk. Our aim was to provide insight into the feasibility, safety and outcomes of RA-MIDCAB, with particular focus on thoracic complexity.

## Patients and methods

Between December 2022 and June 2024, 52 consecutive patients underwent RA-MIDCAB using the da Vinci Xi robotic platform (Intuitive Surgical, Sunnyvale, CA). The study protocol received approval from the Institutional Review Board (PN 1815) that waived informed consent due to the study’s retrospective nature. Inclusion criteria considered isolated left anterior descending (LAD) stenosis as the sole target for revascularization or as a part of HR strategy for patients with left main or multivessel coronary artery stenosis where the non-LAD targets were deemed suitable for PCI by heart-team consensus. The HR strategy also included patients referred for transcatheter valves procedures (mitral and/or aortic valve disease) where a significant LAD stenosis was found and RA-MIDCAB was considered a suitable revascularization strategy [9]. Exclusion criteria were hemodynamic instability or previous cardiac or left-sided thoracic procedures.

### Endpoints

Primary endpoints were 30-day mortality, conversion to sternotomy and incidence of graft injury. Secondary endpoints included overall follow-up survival, major adverse cardiac and cerebrovascular events (MACCEs, defined as cardiac death, myocardial infarction, repeated revascularization, cerebrovascular (CV) accidents) and the cumulative incidence of postoperative complications such as atrial fibrillation (AF), blood transfusion, renal failure, pleural and pericardial effusion requiring drainage.

### Operative time and data collection

Total operative time was calculated as skin-to-skin time, while others robotic operative times included: overall robotic time (from the first arm movement to the final undocking), robotic docking time (from the first port insertion to the first robotic arm movement), and graft-harvesting time (from the first to the last movement performed during left internal thoracic artery [LITA] skeletonization). These times were retrieved from operative reports and My-Intuitive App records (Intuitive Surgical, Sunnyvale, CA).

### Preoperative imaging

As per institutional protocol, all patients underwent preoperative chest CT scan without contrast dye [10]. These scans were reviewed to assess the course of the LITA and LAD. Notably, the presence of intramural LAD was not considered a contraindication to the robotic approach. Chest CT was used to calculate HI and CR.

- HI was calculated by dividing the transverse diameter of the chest (maximum width) by the anterior–-posterior distance (the smallest distance between the anterior surface of the vertebral body and the posterior surface of the sternum). An HI < 2.0 was considered normal, while values of HI > 2.0 suggested progressive reduction of the antero-posterior chest diameter, which may be associated with complex anatomy like pectus excavatum [11].
- CR was defined as the ratio of the maximal horizontal cardiac diameter to the maximal horizontal thoracic diameter (measured from the inner edge of the ribs to the edge of the pleura). A CR between 0.42 and 0.50 was considered normal, while CR > 0.50 were considered indicative of reduced maneuvering space in the chest due to a widened cardiac shape [12].

Based on these criteria, HI > 2.0 and/or CR > 0.50 were regarded as markers of increased “chest complexity” which could potentially impact the feasibility and outcomes of minimally invasive robotic-assisted CABG.

### Surgical technique

As previously described [2], the RA-MIDCAB procedure consists of two main steps: (I) robotic harvesting of the LITA and (II) direct LITA-to-LAD anastomosis through a small thoracotomy (4–5 cm).

### Robotic LITA harvesting (Step I)

Three 8-mm ports were introduced into the left hemithorax: (i) a working port at the II intercostal space along the midclavicular line, (ii) a 30° camera port at the IV intercostal space along the midclavicular line, and (iii) another working port at the VI intercostal space along the anterior axillary line.

AirSeal™ technology (Intuitive, Sunnyvale, CA, USA) was used to insufflate CO_2_ into the chest cavity and to evacuate smoke, improving both exposure and visualization. The triangulation between the camera and working ports was carefully adjusted to optimize the degree of dexterity and avoid conflicts between the robotic instruments (docking phase). A subphrenic pericardiotomy (2–3 cm) was then performed robotically, evacuating any pericardial fluid. Next, the anterior fat pad was removed, and the pericardium was widely opened, starting above the pulmonary artery and extending toward the xiphoid process. Once the LAD was identified, the harvesting of the LITA began.

The LITA was harvested in a skeletonized fashion by dividing the artery from the surrounding satellite veins and arterial side branches using micro-bipolar forceps. A monopolar low-energy spatula was used for finer dissection as needed. After administering heparin, the LITA was divided and fixed to the pericardium to promote hemostasis and fast recovery.

### LITA-to-LAD anastomosis (Step II)

To optimize the positioning of the mini-thoracotomy, the 30° camera was inverted upwards, and a skin incision was made directly above the LAD target. The second surgical phase proceeded in standard fashion. The robotic arms were undocked, and the trocars were removed from the chest. A 4-cm mini-thoracotomy was performed at the IV intercostal space. The incision was adjusted anteriorly or laterally based on the visualization of the LAD during the docking phase. After suspending the pericardium (which was previously opened robotically), a heart stabilizer (Octopus Nuvo™, Medtronic, Minneapolis, MN, USA) was inserted through the VI intercostal space port. The LAD was firmly stabilized, allowing for optimal visualization and manipulation during the anastomosis. A 2–3-mm arteriotomy was performed on the LAD, followed by insertion of a coronary shunt. The LITA-to-LAD anastomosis was then performed using a running Prolene 8/0 suture.

Before administering protamine, the anastomosis was thoroughly checked for leaks and optimal flow with a transit time flow measurement (TTFM) device (MiraQ Cardiac, Medistim, Oslo, Norway).

### Closure

A single chest tube was inserted into the left pleural space for drainage. The fat pad above the pericardium was sutured back in place to protect the distal anastomosis. Finally, the mini-thoracotomy was closed in standard fashion.

### Statistical analysis

The database was formatted through Microsoft-Excel® 365 software (Microsoft, Redmond, Washington) and later imported to the IBM-SPSS® software ver.29.02 (IBM SPSS Inc. Chicago, Illinois); the use of the Stata® software ver.18.5 (Stata Corporation, College Station, Texas) and R ver.4.4.1 (R Project for Statistical Computing, Vienna, Austria) in RStudio were also considered. Normality of the distributions was assessed using the Kolmogorov––Smirnov test. Continuous variables were presented as means ± SD for normal distribution, or median and min/max for skewed distributions, and compared with the use of Student’s T-test or the Mann––Whitney, accordingly. Correlations were evaluated with the Pearson’s or Spearman’s rank test. Categorical variables were presented as frequencies and percentages and compared with the Chi-Squared test or the Fisher’s exact test, as appropriate; associations of the crosstabs were verified using standardized adjusted residuals. Univariable and multivariable logistic regressions were run to study the relationships among dependent and independent variables. A trend line was fit to the robotic time data using LOESS (locally estimated scatterplot smoothing) to visually depict the temporal trend in operative times across patients. LOESS smoothing is a non-parametric method that fits local regressions to subsets of the data, providing a flexible trend line that can capture non-linear patterns in the temporal changes. To assess the statistical significance of the temporal trend, a linear regression model was applied. A two-sided alpha level of 0.05 was used for all tests.

## Results

Patients’ baseline characteristics and thoracic biometric parameters are depicted in Table 1. Robotic LITA harvesting was successfully completed in 98.1% (51/52) of patients. One patient had proximal LITA injury due to at an early phase of the endoscopic harvesting (case #27) and requiring abortion of the surgical procedure due to a lesion impossible to repair robotically. Bail-out strategy for this case was PCI to LAD. Thus, 51 successful LITA take-down and LAD anastomosis were performed. Operative times are reported in Table 2. All patients underwent flow measurement at the end of the surgical procedure before proceeding with protamine administration. Mean measured graft flow was 19.9 ± 8.5 mL/min with a mean distal pulsatility index (PI) of 2.8 ± 0.6. Only one patient required intraoperative anastomosis revision for high PI values with successful recover after re-completing the anastomosis. In this patient, peripheral assistance with extracorporeal circulation was required for ventricular arrhythmia during the second cardiac manipulation.

**Table 1:** Patients baseline characteristics and thoracic indexes

Preoperative yariables	
No of patients	52
Age	68.5 ± 11.5
Female	9 (17.3%)
Hypertension	48 (92.3%)
Diabetes	17 (32.7%)
Dyslipidemia	46 (88.5%)
Smoker	13 (25%)
Former smoker	34 (65.4%)
BMI	26.2 ± 4.4
Obesity	11 (21.2%)
COPD	8 (15.4%)
Chronic renal failure	12 (23.1%)
Creatinine	1.0 (0.4–8.1)
Hemodialysis	4 (7.7%)
CV events	7 (13.6%)
Extracardiac arteriopathy	11 (21.2%)
Atrial fibrillation	5 (9.6%)
Myocardial infarction	19 (36.5%)
Previous PCI	23 (44.2%)
EF	56 (20–75)
PAPs	28.6 ± 7.5
PAPs > 30	14 (26.9%)
EuroSCORE II	1.5 (0.5–11)
Coronary disease	
Left main	19 (36.5)
LAD	51 (98.1%)
Cfx	25 (48.1%)
RCA	26 (50%)
Thoracic parameters	
Cardiothoracic Ratio	0.50 ± 0.05
Haller index	2.0 ± 0.3
Difficult chest	32 (61.5%)
Very difficult chest	12 (23.1%)

*BMI* body mass index, *CV* cerebrovascular, *PCI* percutaneous coronary intervention, *EF* ejection fraction, *PAPs* pulmonary artery pression systolic, *LAD* left anterior descending, *Cfx* circumflex, *RCA* right cornary artery

**Table 2:** Operative times

Operative parameters	Minutes
Total operative time	180 (65–300)
Docking time	13 (5–40)
Overall robot time	63 (30–112)
Graft harvesting time	43.7 ± 10.5

RA-MIDCAB surgery was part of a hybrid strategy in 18/52 patients (34.6%). Among those, 3 patients had surgical revascularization performed after recovering from a percutaneous valve procedure. Besides, 11/15 RA-MIDCAB (73.3%) were performed before the PCI. Thereafter, when angiographically investigated at the time of the PCI, all LITA-LAD grafts were patent without evidence of LITA injury or significant narrowing.

In this preliminary experience of 52 cases, perioperative incidence of mortality and MACCEs was 0%. No patients required conversion to sternotomy at any phase of the surgical procedure, while the incidence of irreversible graft injury was 1.9%. Cumulative incidence of postoperative complications was 38.5% (20/52), details and distributions are depicted in Table 3. Of note, among the seven patients experiencing acute renal failure, five were patients with preoperative chronic renal failure.

**Table 3:** Perioperative details and complications

Perioperative details	
*Intraoperative blood loss (mL)*	50 (10–700)
ICU stay (hours)	20 (14–116)
Mechanical ventilation (hours)	4 (0–10)
Complications	
Graft injury	1 (1.9%)
Atrial fibrillation	10 (19.2%)
Blood transfusion	3 (5.8%)
Pleural effusion	2 (3.8%)
Pericardial effusion	0%
‍ Acute kidney injury	7 (13.5%)
‍Postoperative details	
‍ Chest tube loss after 24 h (mL)	300 (range: 0–1450)
‍ Chest tube loss at removal (mL)	415 (range: 10–2180)
‍ Length of stay (days)	7.3 ± 1.3
‍ Discharged home	31 (59.6%)

*ICU*  intensive care unit

**Fig. 1:**
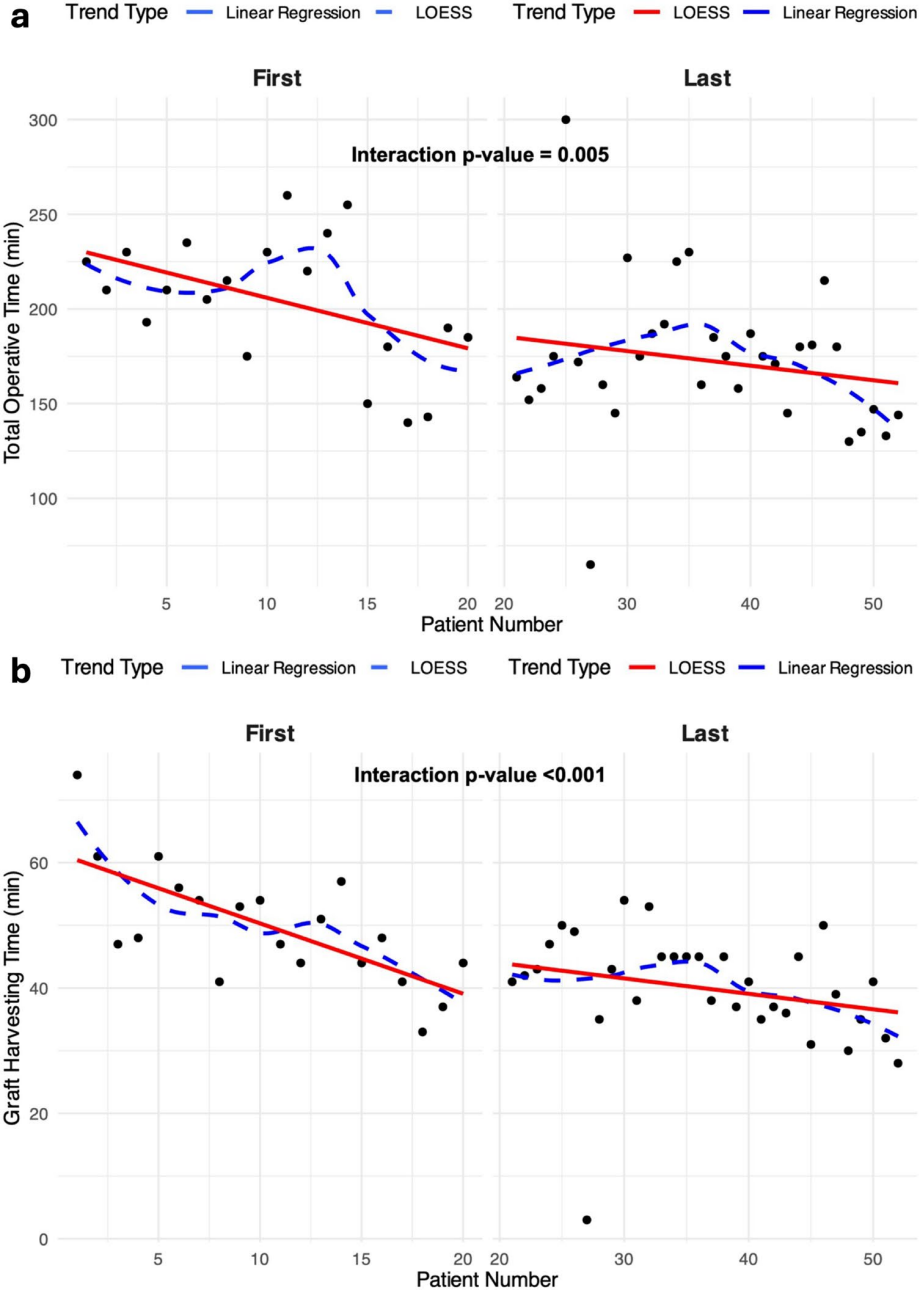
Linear regression analysis between progressive patient number and total operative time (**A**) and graft-harvesting time (**B**). Caption: *LOESS* locally estimated scatterplot smoothing

**Fig. 2:**
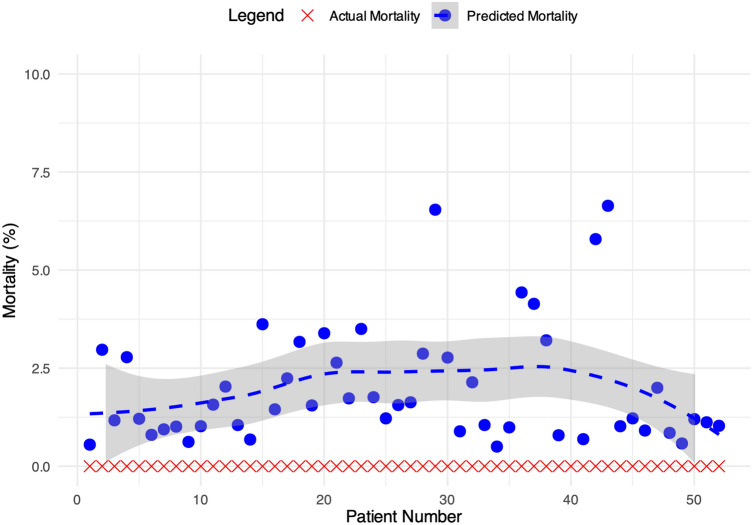
Relationship between expected and observed mortality. Caption: *LOESS*  locally estimated scatterplot smoothing

Spearman’s rho analysis did not show any significant correlation between BMI, EuroSCORE II, HI, CR with total robotic, docking and graft-harvesting times as shown in Supplemental Table 1. Backward conditional logistic regression demonstrated that cumulative incidence of postoperative complications did not correlate with operative robotic times, BMI and thoracic indexes. We therefore classified patients with at least one parameter of chest complexity (either HI or CR) as “difficult chest” (32/52; 61.5%), and those patients with both as “very difficult chest” (12/52; 23.1%). The presence of a “difficult” or “very difficult” chest did not correlate at logistic regression analysis with the incidence of postoperative complications (Supplemental Table 2). Linear regression analysis investigating performance variability across experience progression (all-comers from 1 to 52) showed a significant reduction of the total surgical, total robot and graft-harvesting times (*p* < 0.05), while docking time did not (*p* = 0.107). Conversely, no significant deviations were reported when operative times were correlated with increasing values of BMI, EuroSCORE II, CR and HI, with exception of the docking time related with HI (*p* < 0.05) and the graft-harvesting time related to the CR (*p* < 0.05). Both showed a negative temporal trend with increasing values of HI and CR that suggest a more complex anatomy (Supplemental Figs 1- 20). Lastly, linear regression analysis was performed by dividing the overall population into two groups: a significant change in trend (*p* < 0.001) was found after the case #20 either analyzing the total operative time (Fig. 1A) as well as the graft-harvesting time (Fig. 1B). Figure 2 shows relationship between expected and observed mortality being this latter below the threshold. Median follow-up was 13.4 months (2.1–23.4) with 0% of mortality or incidence of MACCEs.

## Comment

This preliminary analysis of 52 cases of RA-MIDCAB showed: i) RA-MIDCAB is safe and effective minimally invasive option for coronary artery revascularization, ii) excellent clinical outcomes with low complication rate can be achieved even in the early stage of the surgical learning curve; iii) operative times were not influenced by patients’ anatomic factors, and longer surgical duration did not correlate with an increased incidence of postoperative complications, suggesting that robotic surgery in patients with difficult chest anatomy does not lead to longer operative times or higher complication rates; iv) reproducibility of surgical performance was achieved even at the early stage of the learning, regardless of patients complexity or challenging chest anatomy.

In Europe, there has been a gradual increase in the use of robotic-assisted cardiac procedures over the past decade, yet skepticism remains, particularly regarding the safety of the procedure in the early stages of the learning curve [7]. Concerns about long operative times also discourage cardiac teams from initiating robotic programs. However, evidence from larger series suggests significant improvements in surgical performance within the first ten cases, with full competence typically reached after around 50 cases [13, 14]. In our series, excellent results were achieved early on, with low mortality, a minimal incidence of MACCEs, and a low rate of graft injury. Importantly, no formal patient selection criteria were applied in our early cases, meaning a range of patients with different BMI, EuroSCORE II scores, and chest wall anatomies were included, even in the first ten cases. For instance, seven out of the first ten patients had chest anatomy deemed “difficult” based on positive CR and/or HI. We may hypothesize that previous experience in minimally invasive off-pump coronary artery revascularization surgery positively influenced our clinical results. Indeed, the robust experience in MIDCAB surgery allowed our group to focus our robotic training only on step I (robotic LITA harvesting), thus reducing the length of a more complex learning curve in which a double innovation is introduced in the same setting such as robotic harvesting (I) and minimally invasive beating heart revascularization surgery (II). Safety and efficacy profile of this stepwise approach have been previously investigated [15]. Interestingly, Spearman’s analysis showed no direct correlation between operative times and BMI or chest parameters. Moreover, backward logistic regression did not correlate postoperative complications with operative times and thoracic indexes. In the case of adverse event #27, which involved irreversible graft injury, the patient was a fragile, obese individual with long-term steroid use for rheumatoid disorder. The operator opted to abort the procedure after the learning phase for bail-out right ITA harvesting had not yet been completed, transferring the patient to the cath lab for PCI. This highlights the importance of surgical experience and decision-making during early cases.

When compared to other high-volume centers for robotic cardiac surgery, our results demonstrate a lower incidence of death, conversion to sternotomy, and MACCEs. These findings align with studies like those by Halkos et al., who analyzed outcomes between the first 500 cases (a threshold for achieving competency) and the second 500 cases (indicating mastery). In their cohort, overall mortality was 1% (5/500), with only 2 cardiac-related deaths [3]. Similarly, Bonatti et al. reported no hospital mortality in their initial experience with 50 cases, while a later series of 226 patients had an early mortality rate of 1.3% [15, 16]. Graft injury in their series was reported in 1.8% of cases, which is consistent with our findings.

The median EuroSCORE II for our cohort was 1.5 (range 0.5–11), suggesting a low-intermediate risk population. The RA-MIDCAB approach, particularly in hybrid settings, appears to have had a positive impact on perioperative mortality (Fig. 2), although the small sample size of our cohort may limit the power to detect this effect definitively.

The overall incidence of postoperative complications was 38.5% (20/52), consistent with other robotic series, and logistic regression analysis failed to identify a relationship between operative times, chest wall parameters, and complication rates. This suggests that complications are not associated with longer operative times, regardless of the patient's thoracic complexity. Notably, there was no increase in the mean length of stay, although other centers using fast-track protocols report a slightly shorter stay. We intentionally avoided including patients in a fast-track protocol, as this approach is not yet standardized at our institution. While most cardiac surgery patients in our institution are discharged to rehabilitation, 59.6% (31/52) of patients were discharged home directly.

Analysis of surgical performance using linear regression charts showed that: i) overall procedural time, robotic time and graft-harvesting time decreased with increasing in surgical experience of the whole surgical team; ii) graft-harvesting time consistently decreased when compared with progressively increased values of unfavorable CR while remained not significant regardless of BMI or HI; iii) robot docking time, showed a tendency of a progressive reduction as the surgical team gained experience and conversely, showed a non-significant positive trend when correlated with progressive high values of BMI; iv) robot time showed no significant variations when correlated with unfavorable BMI, HI or CR, while consistently decreased over the course of the learning curve. Moreover, a comparison between the first 20 cases and the subsequent 32 revealed a significant reduction in total operative time and harvesting time (p < 0.001). This suggests that a breakpoint in our learning curve in terms of operative performances was reached after 20 cases after which the slope became less steep (Fig. 1). These results were confirmed by Oosterlinck et al., where a significant stabilization of their learning curve was found after 25 cases with a significant reduction of complications after 50 cases, although all these studies did not correlate their findings with patients’ anatomy [17]. Although a significant decrease of all trends was predictable with the progression of the learning curve, the stability of the graft-harvesting time with different BMIs and unfavorable anatomies may suggest the use of a robot technology can potentially mitigate drawbacks related to the application of direct vision mammary artery harvesting techniques in a minimally invasive CABG.

Interestingly, when analyzing the docking phase performance in relation to BMI, we observed a tendency to variability at both ends of the BMI spectrum, suggesting that extremely obese or extremely slim patients may present challenges for early-phase surgeons. Of note, more than 50% of the study population (27 of 52) predominantly fell within the overweight range (> 25 kg/m^2^), and around 25% (12 of 52) were overweight (> 30 kg/m^2^) or underweight (< 20 kg/m^2^). However, these findings further support the idea that robotic surgery can reduce variability and lead to consistent surgical performance once experience is gained. Our data suggest that patient’s chest complexity may increase rapidly as robotic cardiac surgery programs gain experience, with initial case selection being crucial for safety, but more challenging cases becoming feasible as proficiency improves.

This study has some limitations mainly due to the retrospective nature and the small sample size. Moreover, being a single-center study performed by a single surgeon, results may not be generalizable. TTFM interpretation may be limited by the lack of degree of coronary stenosis and systemic blood pressure at the time of measurement. Finally, potential selection bias exists, particularly concerning the initial patients undergoing the procedure, as they may have been selectively chosen based on clinical factors which could impact the applicability of the outcomes to a wider patient population. Being this an early experience, a more strict selection of patients may have been performed, despite attempts to minimize it based on specific inclusion and exclusion criteria.

In conclusion, we believe that the robotic approach offers reproducible and safe outcomes, even in difficult chest anatomies, as demonstrated by the consistency of surgical performance across cases independently from surgical risk. However, the success of a robotic program hinges on the development of a robust training structure, which includes simulation and proctoring. We have invested significant time in simulation training for surgeons, nurses, and anesthesiologists, totaling over 100 h of training across all team members. The use of tele-mentoring, further underscores the potential for remote support in enhancing the learning curve. Building a strong robotic cardiac program requires coordinated effort and continued refinement of skills at every level.

## Supplementary Information

Below is the link to the electronic supplementary material.

Supplementary file1 (DOCX 518 KB)

Supplementary file2 (DOCX 21 KB)

## Data Availability

The datasets generated during and/or analyzed during the current study are available from the corresponding author on reasonable request, pending institutional approval.
