# Supplementary material for: Patient complexity does not affect surgical learning curve and clinical outcomes during early experience in robotic assisted coronary surgery
Source: J Robot Surg. 2025 May 28;19(1):245. doi: 10.1007/s11701-025-02370-w (PMC12119776; doi:10.1007/s11701-025-02370-w)
Supplement: Supplementary file 1 — Supplementary file1 (DOCX 518 KB) [file 11701_2025_2370_MOESM1_ESM.docx]

Supplemental Figure 1 – Linear regression analysis between BMI and docking time

BMI = body mass index; LOESS = locally estimated scatterplot smoothing

Supplemental Figure 2 – Linear regression analysis between BMI and graft harvesting time

BMI = body mass index; LOESS = locally estimated scatterplot smoothing

Supplemental Figure 3 – Linear regression analysis between BMI and total operative time

BMI = body mass index; LOESS = locally estimated scatterplot smoothing

Supplemental Figure 4 – Linear regression analysis between BMI and total robotic time

BMI = body mass index; LOESS = locally estimated scatterplot smoothing

Supplemental Figure 5 – Linear regression analysis between CR and docking time

CR = cardiothoracic ration; LOESS = locally estimated scatterplot smoothing

Supplemental Figure 6 – Linear regression analysis between CR and graft harvesting time

CR = cardiothoracic ration; LOESS = locally estimated scatterplot smoothing

Supplemental Figure 7 – Linear regression analysis between CR and total operative time

CR = cardiothoracic ration; LOESS = locally estimated scatterplot smoothing

Supplemental Figure 8 – Linear regression analysis between CR and total robotic time

CR = cardiothoracic ration; LOESS = locally estimated scatterplot smoothing

Supplemental Figure 9 – Linear regression analysis between EuroSCORE II and docking time

LOESS = locally estimated scatterplot smoothing

Supplemental Figure 10 – Linear regression analysis between EuroSCORE II and graft harvesting time

LOESS = locally estimated scatterplot smoothing

Supplemental Figure 11 – Linear regression analysis between EuroSCORE II and total operative time

LOESS = locally estimated scatterplot smoothing

Supplemental Figure 12 – Linear regression analysis between EuroSCORE II and total robotic time

LOESS = locally estimated scatterplot smoothing

Supplemental Figure 13 – Linear regression analysis between HI and docking time

HI = Haller Index; LOESS = locally estimated scatterplot smoothing

Supplemental Figure 14 – Linear regression analysis between HI and graft harvesting time

HI = Haller Index; LOESS = locally estimated scatterplot smoothing

Supplemental Figure 15 – Linear regression analysis between HI and total operative time

HI = Haller Index; LOESS = locally estimated scatterplot smoothing

Supplemental Figure 16 – Linear regression analysis between HI and total robotic time

HI = Haller Index; LOESS = locally estimated scatterplot smoothing

Supplemental Figure 17 – Linear regression analysis between patient number and docking time

LOESS = locally estimated scatterplot smoothing

Supplemental Figure 18 – Linear regression analysis between patient number and graft harvesting time

LOESS = locally estimated scatterplot smoothing

Supplemental Figure 19 – Linear regression analysis between patient number and total operative time

LOESS = locally estimated scatterplot smoothing

Supplemental Figure 20 – Linear regression analysis between patient number and total robotic time

LOESS = locally estimated scatterplot smoothing
