# Supplementary material for: Patient complexity does not affect surgical learning curve and clinical outcomes during early experience in robotic assisted coronary surgery
Source: J Robot Surg. 2025 May 28;19(1):245. doi: 10.1007/s11701-025-02370-w (PMC12119776; doi:10.1007/s11701-025-02370-w)
Supplement: Supplementary file 2 — Supplementary file2 (DOCX 21 KB) [file 11701_2025_2370_MOESM2_ESM.docx]

Supplemental Table 1. Spearman’s rho analysis.

|  |  | ***BMI*** | ***EuroSCORE II*** | ***Cardiothoracic Ratio*** | ***Haller Index*** |
| --- | --- | --- | --- | --- | --- |
| ***Total Operative Time*** | Coefficient | 0,059 | -0,271 | -0,136 | -0,101 |
|  | Sig. (2-tailed) | 0,68 | 0,052 | 0,34 | 0,47 |
| ***Docking Time*** | Coefficient | 0,112 | -0,133 | -0,028 | -0,236 |
|  | Sig. (2-tailed) | 0,43 | 0,35 | 0,84 | 0,093 |
| ***Overall Robot Time*** | Coefficient | 0,004 | -0,198 | -0,171 | -0,183 |
|  | Sig. (2-tailed) | 0,98 | 0,16 | 0,23 | 0,19 |
| ***Graft Harvesting Time*** | Coefficient | 0,066 | -0,171 | -0,270 | -0,119 |
|  | Sig. (2-tailed) | 0,64 | 0,23 | 0,053 | 0,40 |

BMI = body mass index

Supplemental Table 2. Logistic Regression on postoperative outcomes

|  | ***Sig.*** | ***Exp(B)*** | ***95% C.I.for EXP(B)*** | |
| --- | --- | --- | --- | --- |
|  |  |  | ***Lower*** | ***Upper*** |
| ***Body Mass Index*** | 0,61 | 0,941 | 0,748 | 1,185 |
| ***EuroSCORE II*** | 0,58 | 1,133 | 0,730 | 1,757 |
| ***Total Operative Time*** | 0,58 | 1,008 | 0,981 | 1,035 |
| ***Docking Time*** | 0,39 | 0,953 | 0,854 | 1,063 |
| ***Overall Robot Time*** | 0,44 | 0,932 | 0,779 | 1,114 |
| ***Graft Harvesting Time*** | 0,94 | 0,993 | 0,821 | 1,199 |
| ***Difficult Chest*** | >0,99 | 1,000 | 0,282 | 3,544 |
| ***Very Difficult Chest*** | 0,93 | 1,071 | 0,250 | 4,591 |
